# Viability Conditions for a Compartmentalized Protometabolic System: A Semi-Empirical Approach

**DOI:** 10.1371/journal.pone.0039480

**Published:** 2012-06-27

**Authors:** Gabriel Piedrafita, Kepa Ruiz-Mirazo, Pierre-Alain Monnard, Athel Cornish-Bowden, Francisco Montero

**Affiliations:** 1 Departamento de Bioquímica y Biología Molecular I, Universidad Complutense de Madrid, Madrid, Spain; 2 Departamento de Lógica y Filosofía de la Ciencia, Universidad del País Vasco, Donostia-San Sebastián, Spain; 3 Unidad de Biofísica, Consejo Superior de Investigaciones Científicas-Universidad del País Vasco, Leioa, Spain; 4 Center for Fundamental Living Technology, University of Southern Denmark, Odense, Denmark; 5 Unité de Bioénergétique et Ingénierie des Protéines, Centre National de la Recherche Scientifique, Marseille, France; Queen Mary, University of London, United Kingdom

## Abstract

In this work we attempt to find out the extent to which realistic prebiotic compartments, such as fatty acid vesicles, would constrain the chemical network dynamics that could have sustained a minimal form of metabolism. We combine experimental and simulation results to establish the conditions under which a reaction network with a catalytically closed organization (more specifically, an (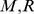)-system) would overcome the potential problem of self-suffocation that arises from the limited accessibility of nutrients to its internal reaction domain. The relationship between the permeability of the membrane, the lifetime of the key catalysts and their efficiency (reaction rate enhancement) turns out to be critical. In particular, we show how permeability values constrain the characteristic time scale of the bounded protometabolic processes. From this concrete and illustrative example we finally extend the discussion to a wider evolutionary context.

## Introduction

By means of a complex set of interconnected enzymes, living systems have mastered the coupling and kinetic control of chemical reactions leading to robust forms of cyclic self-production, generally conceived as metabolisms. Biochemistry, however, takes place within compartments. All known metabolisms are *vectorial* [1]: they involve gradients, processes occurring in compartmentalized space, diffusion and transport of compounds across diverse boundaries, all of these being deeply entangled with enzyme-regulated chemical pathways. The complementary relationship established between a network of reaction processes and its physical-topological border (most distinctively, the cytoplasmic membrane) has often been highlighted as a central aspect of biological organization, and even considered as the defining feature of life [2], [3]. However, despite the claims of the “compartment-first” school of thought in the origins of life research field [4]–[9] (see also [10]), there have been few empirical or theoretical studies of the actual conditions for viability of that kind of system (see Note 1 in Text S1), i.e. about the mutual physical-chemical constraints that a minimal cyclic reaction network (a protometabolism) and a boundary (e.g. a prebiotic lipid vesicle) impose on each other.

Until recently, self-assembling compartments (in particular, topologically closed lipid bilayers, or vesicles) have been regarded as a challenge for the development of any complex chemistry, due to the barrier to the free diffusion of solutes they represent and the corresponding reduction of the molecular precursor accessibility to the inner aqueous core of the system. Therefore, some authors have shown preference for a scheme of prebiotic transitions in which (bio-)chemistry develops without lipid compartments [11]–[13] or, at most, in their vicinity [14], [15]. Recent experimental work on protocell systems with membranes made of mixtures of fatty acids and other prebiotically plausible amphiphiles [16], [17] has shown, however, that vesicles do not necessarily constitute such impermeable barriers [18], particularly for non-ionic and low-molecular-weight compounds. From these new pieces of evidence, an alternative *co-evolutionary* scenario can be envisioned in which reaction networks would very early be hosted within protocell compartments, becoming increasingly both interdependent and complex thereafter.

The role of lipid phases and compartments that likely preceded bio-membranes must have gone beyond their primary anti-dilution effects of preventing the irreversible loss of soluble, non-abundant–but often essential–organic compounds. For example, lipid bilayer and multilayer structures have been reported to assist polymerization of both nucleic acids and peptides in various experimental conditions [19]–[23], or to harbor light energy transduction mechanisms [24]. In addition, encapsulating a reaction network (in particular, a network comprising populations of replicating molecules) within a vesicle with potential, itself, for reproduction (as a whole system) has often been underlined as a key evolutionary step towards living organisms [25], [26]. Nevertheless, many other possible advantages of combining aqueous and lipidic faces (i.e. *soft* interfaces; see Note 2 in Text S1) deserve both theoretical and empirical explorations. If one recognizes the far-reaching effects that simple spatial diffusion has on chemical processes (e.g. spontaneous pattern formation [27]), it is then difficult to overlook the potential of self-assembling compartments to modulate chemistry (e.g. through more precise control of diffusion rates) or even to enable “new chemistries” (unexpected in open solution conditions). As an illustrative example, we can mention how the use of water-in-oil micro-emulsions to adjust the diffusion rates of the different compounds involved in an oscillating reaction system, such as a Belousov-Zhabotinsky reaction system, has allowed the empirical observation of a very diverse range of complex spatial-temporal patterns of chemical behavior [28].

In this context, it would be of great interest to determine the conditions for viability of cyclic reaction networks (e.g. coupled autocatalytic loops with potential for self-maintenance) within vesicles made of prebiotically plausible amphiphilic molecules. Although several interesting results have been recently obtained on those lines (e.g. the achievement of the autocatalytic formose reaction system in vesicles, even if these were not prebiotic [29]), bottom-up approaches to this general problem are rare. In contrast, some theoretical studies have progressed towards a rigorous definition and characterization of metabolism. For instance, it has been argued that any metabolism must be based on autocatalysis, through coupled autocatalytic reaction networks [30], [31] or reflexively autocatalytic sets [32], [33]. In addition, authors such as Maturana and Varela [3], Rosen [34] or Fontana and Buss [35] consider that metabolic behavior requires an operationally-closed organization. Thus, protometabolisms must have developed mechanisms of self-production, such as the ability to produce their own catalysts, achieving catalytic closure [36]. In these theoretical approaches, however, the compartment is frequently disregarded or treated in terms that are too abstract.

Here we attempt to fill that gap, combining theoretical and experimental results obtained through various methods into a common framework of analysis. Specifically, we explore the implications of inserting a cyclic network of reactions with a self-productive and self-repair architecture [37]–[39], into prebiotically plausible fatty acid vesicles. We will pay special attention to the way in which the properties (in particular the permeability) of these initial, protocellular compartments could impose limits on the actual rates of the reactive processes subject to encapsulation, if the system is to avoid self-suffocation [31].

## Results

### Model Description

**Figure 1:**
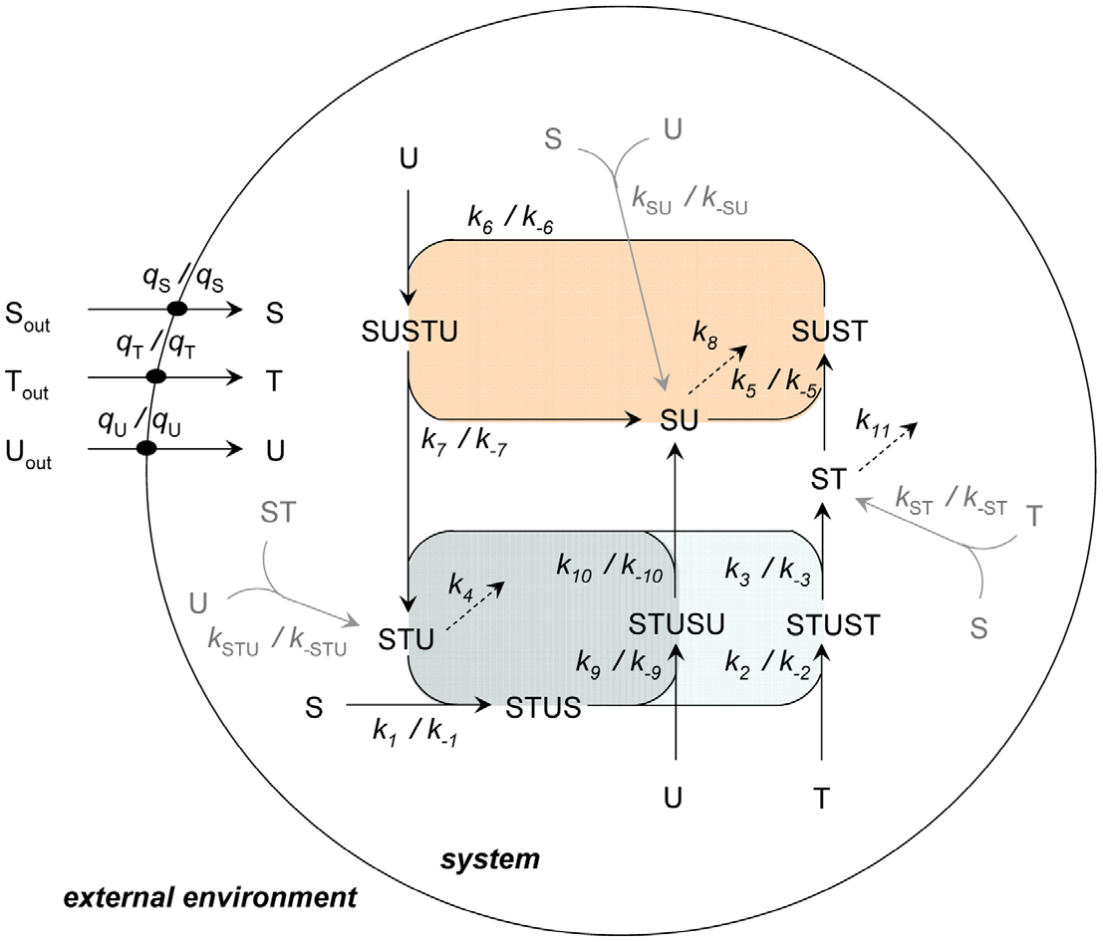
Kinetic model of a compartmentalized protometabolic network. Two different domains are defined: an *external environment*, considered as an unlimited source of precursor molecules (S, T and U); and a *system* in which a set of protometabolic processes take place, encapsulated by a semipermeable membrane (represented as a sphere). Its boundaries allow for the passive diffusion of the precursors into the inner aqueous core. Three intertwined catalytic cycles, highlighted in different colors, define the *core protometabolism* (reactions depicted as black arrows), transforming the precursors into catalysts STU, ST and SU; whereas three accessory reactions, shown in light grey, correspond to the non-catalyzed formation of these catalysts, which are also irreversibly degraded through steps 4, 8 and 11 (dashed arrows). The concentrations of the distinct species inside the system are treated as variables, and all processes except the degradation reactions are reversible. Forward rate constants are denoted 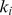, adjacent to their corresponding processes (direction of the arrows), while reverse rate constants, when pertinent, appear as 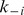. The nutrients S, T and U can diffuse through the membrane in both directions: the same rate constants 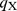 are assumed for their incorporation and efflux.

The protometabolic model we will explore (Fig. 1) consists of two distinct domains. First, there is an *external environment*, taken to be an unlimited reservoir of energy-rich precursors (S, T and U), with concentrations that are assumed to have fixed values in that medium (in other words, they are assumed to be buffered by a surrounding chemistry, as in a primordial-soup hypothesis). In addition, there is a *system* containing a protometabolic reaction network limited or enclosed by a semi-permeable primitive membrane. With these premises, the membrane is considered to be permeable to the small precursors, but impermeable to the bulkier metabolic intermediates produced by the internal reaction network [16], [40]. This allows the acquisition (by passive diffusion) of the precursors from the external environment, which is a necessary process for feeding the internal set of reactions, while preventing the leakage of the intermediates. Regarding the internal protometabolic network, it follows a previous model of a simple catalytically-closed metabolism [36], [41], which has been adapted by introducing some slight but non-trivial modifications (in particular, the non-catalyzed, thermodynamically driven, slow production of the main catalysts in the system). The core reaction scheme is composed of three intertwined catalytic cycles (colored in Fig. 1) that are together capable of counteracting the spontaneous degradation of the catalysts STU, ST and SU–shown by the dashed arrows in the graph. Self-maintenance thus results from a trade-off between modest degradation rates and an efficient coupling of condensation reactions that involve the three energy-rich precursors, which essentially provides the system with its own means of production of the degrading catalysts.

As mentioned at the end of the Introduction, the metabolic model has already been analyzed in homogeneous conditions [38], [39] revealing an interesting dynamic behavior, with a stationary state of non-null concentrations of intermediates that proved robust for a range of degradation rates. While S, T and U were then assumed to have constant concentrations, these parameters will be variable now, as the reaction domain is compartmentalized. This constraint is thus translated to the concentration of precursors in the outer, external environment (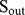, 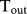 and 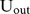). In this way, the impact of membrane permeability on the dynamics of the system can be studied. Additionally, accessory reactions for the non-catalyzed formation of catalysts from precursors have now been included. These uncatalyzed processes are illustrated with grey arrows in Fig. 1, to distinguish them from the rest of internal protometabolic reactions (catalytic transformations plus irreversible degradations) that will be called the “core protometabolism” (black arrows).

All rate constants are treated as invariant, with the fixed values shown in Table 1, except for the degradation rate constants, 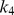, 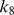 and 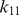, which are varied in the range 0.0–0.6, and the rate constants for the incorporation of precursors, 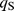, 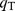 and 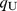, which are varied in the range 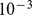
–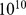. Both the rate constants and the concentrations are assigned consistent units: concentrations are expressed in mM, whereas time is expressed in arbitrary units (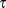) in the absence of strong arguments supporting a particular time scale for such prebiotic processes. Later in the paper we shall discuss how large the unit of time should be.

**Table 1:** Values of rate constants and fixed concentrations in the kinetic protometabolic model.

Kinetic constants	
First-order reactions ( 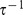 )	Second-order reactions ( 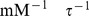 )
*k*_−1_ = 10	*k*_1_ = 100
*k*_−2_ = 10	*k*_2_ = 100
*k*_3_ = 2	*k*_−3_ = 10
0<*k*_4_<0.6	
*k*_−5_ = 1	*k*_5_ = 10
*k*_−6_ = 1	*k*_6_ = 10
*k*_7_ = 0.1	*k*_−7_ = 1
0<*k*_8_<0.6	
*k*_−9_ = 0.05	*k*_9_ = 1
*k*_10_ = 0.05	*k*_−10_ = 0.5
0<*k*_11_<0.6	
*k*_−ST_ = 0.001^*^	*k*_ST_ = 0.02^*^
*k*_−STU_ = 0.001^*^	*k*_STU_ = 0.01^*^
*k*_−SU_ = 0.001^*^	*k*_SU_ = 0.02^*^
Passive diffusion rate constants ( 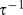 )	
10^−3^<*q_S_*<10^10^ *	
10^−3^<*q_T_*<10^10^ *	
10^−3^<*q_U_*<10^10^ *	
External concentrations (mM)	
[S_out_] = 0.4	
[T_out_] = 0.2	
[U_out_] = 0.1	

All parameters have the same values as already defined in previous work [38], [39], except those marked with an asterisk, which are new.

Notice (from Table 1) that, quite reasonably, the rate constants of uncatalyzed condensation reactions are assigned values significantly smaller than those of the catalyzed processes, to stress the kinetic effect of those primitive catalysts, even if they must have been much less effective in their action than present-day proteins. This was done with care to avoid violating thermodynamic constraints on the global processes, i.e. the equilibrium constant of each one of the uncatalyzed processes is the same as the overall equilibrium constant of the corresponding catalytic cycle forming the same product. Thus, for instance 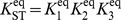 as developed below:
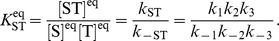



The restriction of constant concentrations of S, T and U in the external environment (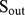, 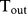 and 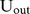) and the exit of degraded compounds (see Note 3 in Text S1) implies a continuous flow of energy and/or matter through the entire system (vesicular system plus its external environment), from the activated precursors, S, T and U, to the waste inactivated products of irreversible degradations. This, together with the condition of irreversibility of the degradation reactions, make the system thermodynamically open, forcing it to operate under non-equilibrium conditions.

### Stationary Solutions of the Model Protometabolism

**Figure 2:**
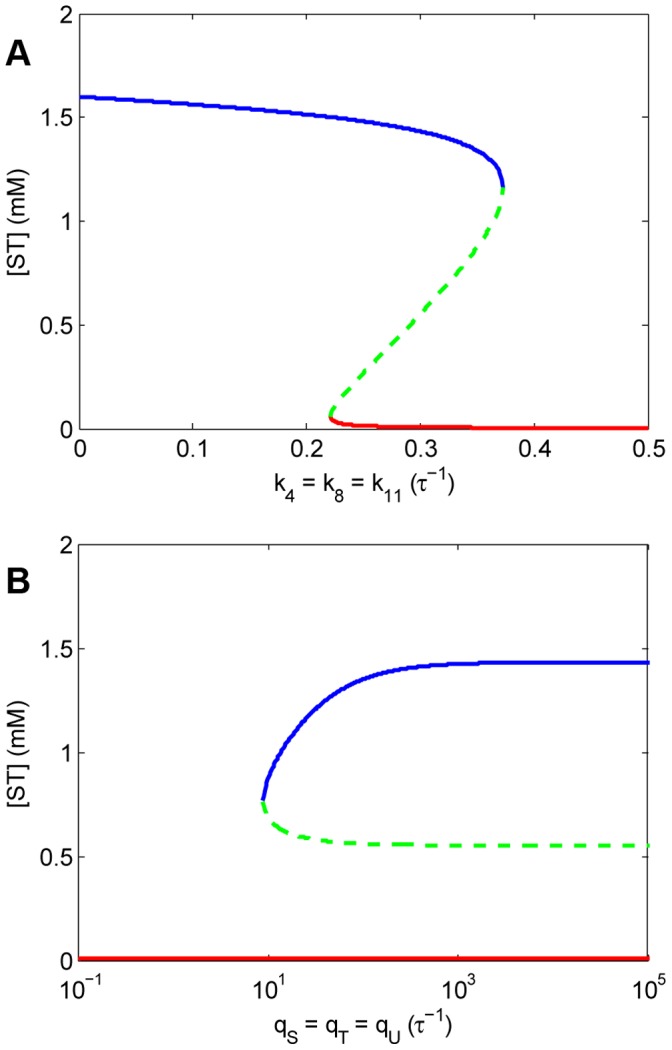
2D bifurcation diagrams. (A) Bifurcation diagram obtained as a function of the values of the degradation rate constants, 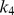, 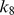 and 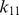, assuming total availability of precursors S, T and U (i.e. instantaneous diffusion). (B) Bifurcation diagram with respect to the values of incorporation rates of precursors, 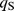, 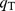 and 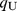, considering degradation rates of 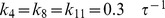. Notice (in B) that for sufficiently large values of 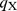, the steady-state concentrations approach those shown in A for 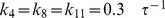. In both (A) and (B) a bifurcation point can be distinguished that separates a region of mono-stability of a residual steady state (with vanishing concentrations of intermediates––depicted in red), from a region of bistability in which that solution coexists with a functional stable steady state with high concentrations of intermediates (in blue), separated by an unstable steady state (a saddle point shown in shaded green). This behavior indicates that both the degradation rate constants and the incorporation rate constants are critical to achieve self-maintenance, given that certain critical thresholds cannot be crossed without loosing that capacity for self-maintenance.

Stationary state solutions (i.e. states with invariant concentrations of intermediates) were calculated (see Materials and Methods) and explored in a wide range of the parameter space. As shown in Fig. 2 and detailed in the text below, the number and type of possible steady states that the system may reach depend both on the values of the degradation rate constants, 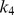, 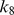 and 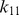, and on the values of the rate constants for the influx of the different precursors, 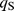, 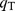 and 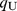. For simplicity, we will only illustrate the effects on the concentration of ST, but qualitatively similar results apply to the behavior of the other intermediate concentrations.

#### Non-limiting diffusion of precursors

We first analyzed the possible steady-state solutions under the extreme assumption of non-limiting diffusion of precursors, i.e. immediate supply of feeding molecules S, T and U. This situation is equivalent to the assumption of instantaneous uptake of precursors from the external reservoir (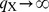), so that the concentrations of precursors are always equilibrated between the external and the internal medium. Therefore, for practical purposes, in this situation we considered the internal concentrations of S, T and U to be fixed and equal to the external ones: 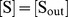, 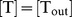 and 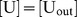. These conditions are similar to those analyzed in previous papers [38], [39] but with the novelty that the uncatalyzed synthesis of the catalysts is now included.

The space of possible steady-state behaviors is mainly sensitive to the degradation rates (Fig. 2a). For relatively high values of degradation rate constants (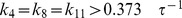) only one steady state is possible, corresponding to a *residual state* (Fig. 2a, shown in red) in which the system shows close-to-zero concentrations of intermediates, mainly due to the slow rates of uncatalyzed production processes. However, when the degradation rate constants are smaller (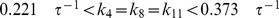), the previous residual state coexists with a non-null steady state with high concentrations of intermediates (Fig. 2a, in blue), which will be referred to as the *functional steady state*. That is, a self-maintaining regime in which the system can keep producing its own components despite the continuous degradation they suffer. In this region of the parameter space, bistability appears: both steady states are shown to be asymptotically stable and appear separated from each other by an *unstable steady state* (a saddle point; Fig. 2a, in green). This third steady state is associated with a separating barrier in the phase space delimiting the attraction areas of the two stable steady states. In this range, therefore, the time evolution of the system towards one state or the other is determined by the initial conditions. Initial concentrations of intermediates over the separating barrier lead to the self-maintaining state, whereas too restrictive conditions, below that separating barrier, lead to the residual state. Finally, when the values of degradation rate constants are even smaller (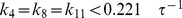), the only steady state present is the non-trivial one corresponding to self-maintenance of large concentrations of intermediates (see Note 4 in Text S1).

Our analysis predicts two bifurcation points (at 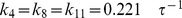 and 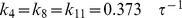) that allow an interesting hysteretic behavior. Assuming that the system is in the residual state, the system remains in the same state as the degradation rate constants decrease (moving to the left in Fig. 2a), as long as the values of the degradation rate constants are greater than the critical point 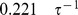. Below this point the system experiences a sudden jump in the concentrations of intermediates to the values in the functional non-trivial stable steady state. Once in this non-trivial stable steady state, the system will tend to remain there, even when the degradation rate constants are increased over the critical point 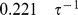 (moving to the right in Fig. 2a). It is necessary to go further to values over the critical point 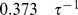 to observe a collapse to the residual state. At this sudden transition all concentrations rapidly become very low, presumably as a consequence of the incapability of the self-producing catalytic cycles to compensate for the fast degradation of its components. This hysteretic behavior is especially interesting because it shows a relatively high robustness of the functional non-trivial steady state with respect to the increase in the values of the degradation rate constants; but also (and perhaps more significantly) because it explains how this system could emerge spontaneously, just from its precursors, given a certain scenario with relatively low values of the degradation rate constants. Indeed, when 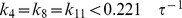, the formation of the functional system becomes certain even if there is no catalyst present initially. The slow, spontaneous uncatalyzed formation of intermediates from precursors will be sufficient to drive the production of a certain amount of catalysts, which in turn triggers the construction of the system. This result represents the first important novelty compared with previous studies along similar lines [38].

#### Rate-limiting diffusion of precursors

Even if primitive membranes were probably much leakier than present bio-membranes [18], it is reasonable to assume a diffusive constraint for nutrient uptake by any encapsulated protometabolism. Every closed membrane, if it is minimally stable, acts as a diffusion barrier, so primitive vesicle bilayers must have imposed kinetic restrictions on the free acquisition of precursor molecules from the external medium. Accordingly, we turn now to study the effects of varying the rate constant for the incorporation of precursors, 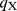, on the system dynamics.

In a first approach, we focused on the steady-state solutions found for diverse values of 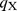 (in the range 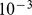
–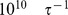), while keeping a fixed value of the degradation rate constants. For instance, Fig. 2b shows the results obtained when 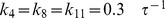. As seen in this figure, for relatively high values of 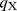 the system is bistable, as for the same degradation parameters with non-limiting diffusion of precursors (Fig. 2a). In fact, not surprisingly, as 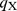 increases the steady-state concentrations approach the values obtained in the analysis with non-limiting diffusion of precursors (i.e. 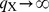). However, the behavior is very different at smaller values of the incorporation rates: the concentrations in the functional steady state (blue line in Fig. 2b) decrease as the values of the rate constants for uptake of precursors progressively decrease, until a critical point is reached (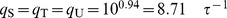) in which the functional steady state merges with the unstable steady state (green line). Below this point the system becomes monostable, with the residual steady state (very small concentrations of intermediates) as the only possible solution (red line in the figure). To avoid its collapse under these conditions, the system must maintain relatively high values of 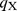; in other words, its membrane needs to be sufficiently permeable.

**Figure 3:**
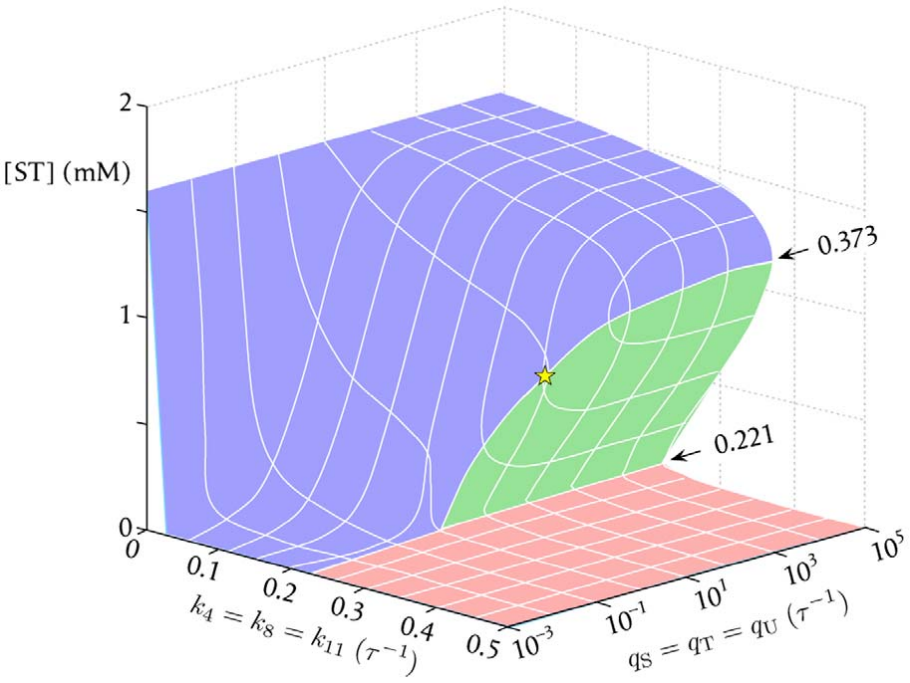
3D bifurcation diagram. Combined bifurcation diagram, showing the dependence of the stationary solutions on both the degradation rate constants (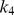, 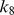 and 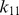), and the rate constants for uptake of nutrients (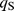, 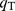 and 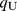). Three different regions are distinguished in the parameter space: (i) For 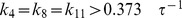, only a residual steady state (red surface) is possible regardless of the values of 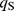, 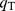 and 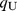. (ii) In the range 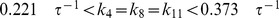 the number of possible attainable steady states is, in contrast, dependent of the values of 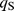, 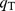 and 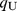. The star shows the location of the critical point obtained for 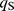, 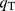 and 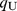 when 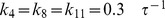. (iii) For 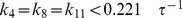, there is again only one possible steady state, regardless of the values of 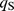, 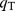 and 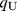. However, 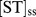 is still strongly dependent on the values of these incorporation rate constants, which remain critical to relate this steady state with a proper functional steady state or, instead, with some residual functioning of the system.

To get a better insight into the general region of the parameter space where the functional non-trivial steady state is permitted, we have extended the previous analysis to other values of 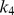, 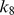 and 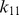, in the range: 0.0–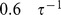. The results are summarized in the three-dimensional bifurcation diagram in Fig. 3. Although the system always finishes at the residual steady state when 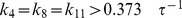, regardless of the values of the incorporation rate constants (i.e. if catalyst degradation occurs too rapidly, then even a high permeability will not prevent the residual state), this set of parameters 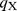 become critical for lower values of the degradation rate constants. In fact, as we saw above, in the range 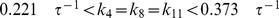 the system becomes bistable. But this only happens for relatively large values of 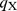: as these values decrease, diffusion cannot completely fulfill the metabolic requirements and intermediate concentrations at the functional non-trivial steady state start to decrease as well (reflected in the curvature of the blue surface in Fig. 3). Following that trend, below a certain critical value 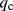, the system suffers a sudden shift to the residual state (edge of the blue surface). This sharp transition occurs for 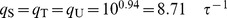 when 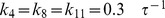 (Fig. 2b, star in Fig. 3), but moves to lower values of 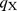 as the degradation rate constants get smaller. There is a parallel decrease in the concentration of ST at which this transition occurs, shown by the progressive fall of the intersection between the blue and green surfaces. This intersection finally merges with the residual state at 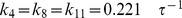 for 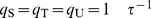.

Below the limit 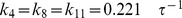 there is no longer a sharp transition between the functional steady state and the residual one, but a continuous space of admissible steady-state concentrations of ST. The system exhibits high concentrations at relatively high values of 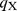, and progressively lower concentrations at more restrictive values of 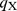. However, except for conditions too close to equilibrium (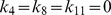), the major decrease in the concentrations is still produced in a narrow range of values of the incorporation rate constants, which is generally between 0.1 and 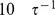. For instance, for 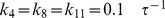 the concentration of ST is 1.29 mM when 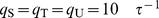, but goes down to 0.13 mM when 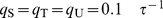. So, even though the system maintains non-null concentrations of intermediates at relatively low values of permeability, they become so small and close to those of the residual steady state that it turns arguable whether the system is functional, *sensu stricto*, under such far-from-optimal conditions. Hence, although strictly speaking a critical value 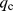 does not exist in the region where 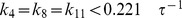, the values of the incorporation rate constants still affect the development of the system into either a functional steady state or a residual state. We will also neglect conditions too close to 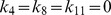, where the independence of 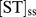 on 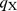 is due to the close-to-equilibrium state of the system (i.e. far from functional conditions).

In conclusion, the rate constants both for the degradation of catalysts (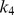, 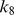 and 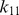), and for the incorporation of feeding molecules (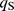, 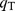 and 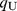) are critical parameters in this model. Thus, in order for the enclosed or compartmentalized protometabolic system to maintain a functional steady state with high enough concentrations of intermediates, it is necessary to consider a relatively limited degradation of catalysts (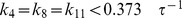) and a relatively high permeability of the compartment to precursors (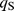, 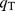 and 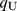 at least in the order of magnitude of 0.1 to 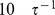).

### Permeability Constraints: an Experimental Survey

**Figure 4:**
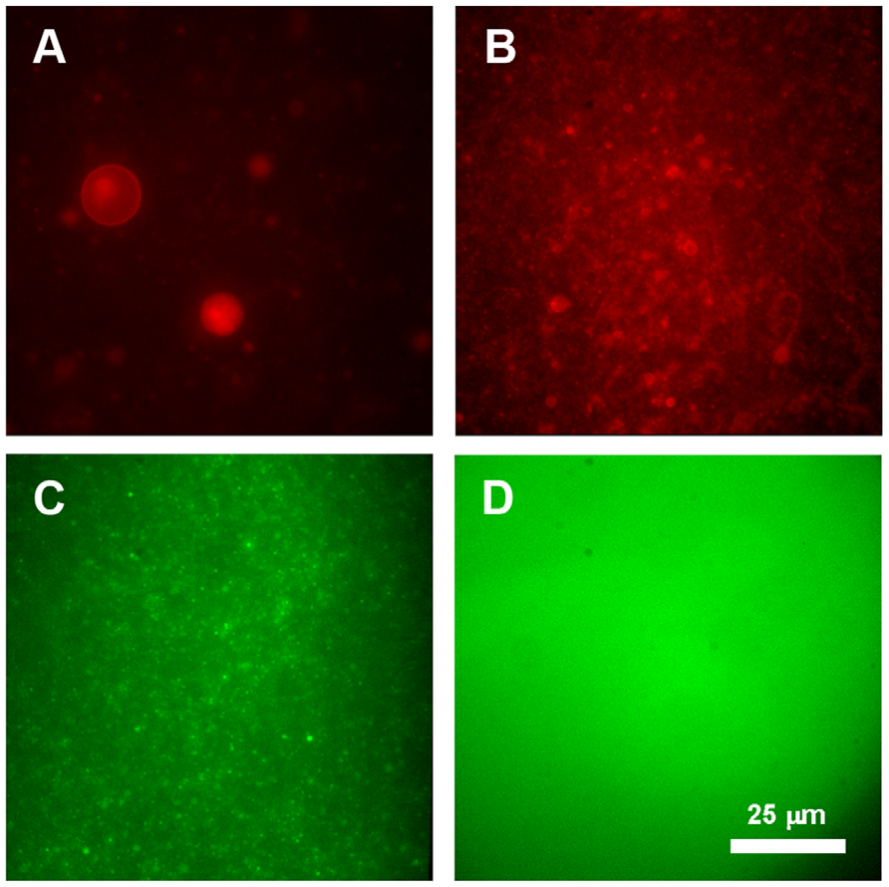
Preparation of fatty acid vesicles encapsulating CF, visualized by epifluorescence microscopy. (A) Micrograph showing the formation of vesicular structures of polydisperse sizes and diverse degree of lamellarity from an aqueous solution of 21 mM LA in 100 mM bicine buffer, pH 7.95. (B) Another LA vesicle suspension, but 1 h after extrusion through polycarbonate filters of 400 nm pore diameter. (C) LA vesicles of monodisperse size just after size exclusion chromatography. Once the non-entrapped material has been removed, vesicles retaining CF exhibit a characteristic green color. (D) The fluorescence spreads throughout the sample after addition of a detergent such as Triton-X100. Samples A and B are stained with the lipophilic dye Nile red. All panels are at the same magnification.

Once the critical effect that passive transmembrane diffusion rates can have on the self-maintenance of this protometabolic system has been shown, it becomes necessary to assess how permeable real primitive lipid bilayers could have been to nutrients. The permeability to a relatively small solute (carboxyfluorescein, CF; see Note 5 in Text S1) was accordingly investigated for two representative experimental compartment models: vesicles made of lauric acid (LA) (a short-chain, fully saturated fatty acid), as a plausible model of a prebiotic, precursor membrane boundary; and vesicles composed of mixed oleic acid and glycerol monooleate (OA/GMO), as a control model for more stable and evolved membrane systems, i.e. “primitive biomembranes” (for a review on the evolutionary aspects of prebiotic compartments see [42]). Before the release experiments were done, the experimental methodology used for encapsulating CF (see Materials and Methods) was validated by fluorescence microscopy (Fig. 4). Both types of amphiphilic systems rapidly formed self-assembled vesicles when prepared at a pH close to the 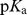 of the fatty acid. In those conditions, samples were also able to encapsulate CF, as confirmed by the observation of small green dots after size-exclusion chromatography (Fig. 4c).

**Figure 5:**
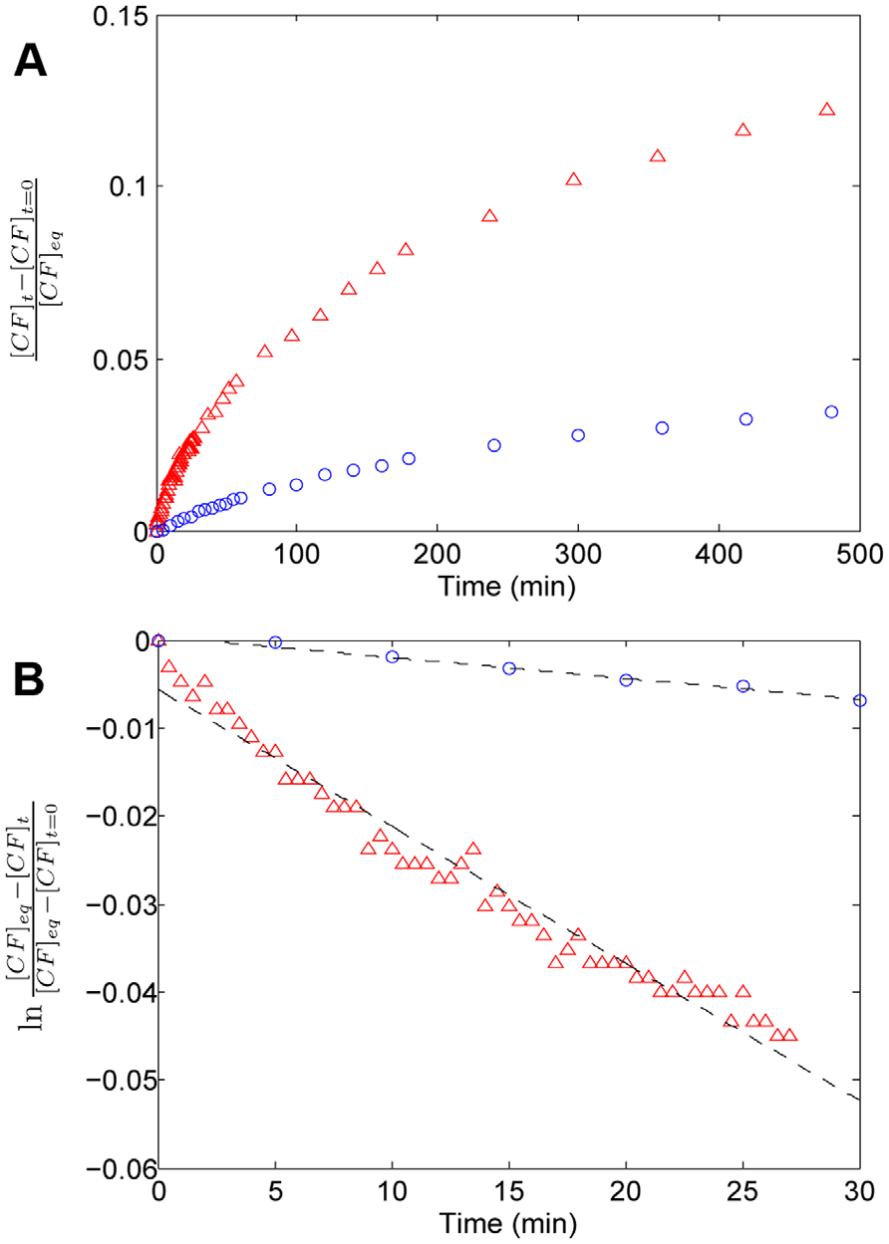
Experimental release profiles of CF from vesicles at 45°C. (A) Time evolution of the concentration of released CF, normalized to the concentration at equilibrium, i.e. after final addition of detergent. The resulting exponential curves were linearized in a semi-logarithmic plot for the first 30 min (B). Linear tendencies are obtained in agreement to Eq. 4 in Text S2, identifying the slope as the rate constant of release 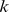. 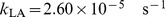 (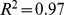); 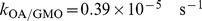 (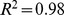). Red triangles: vesicles made of LA. Blue circles: vesicles made of OA/GMO in a molar ratio 2∶1.

Permeability was monitored by measuring solute release at 45°C. Fig. 5a shows the time course of CF liberation from both types of vesicular systems (for convenience, the fluorescence signal has been translated into concentration units using a standard curve; see Text S2). In both cases the data show approximately an exponential decay. As equilibrium could not be reached in either case, even after relatively long waiting times (500 min), a detergent (Triton-X100) was used to solubilize the vesicles and release the encapsulated dye (see Fig. 4d). The resulting values were taken as the reference state 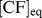, corresponding to the normalized value 1 in the ordinate axis in Fig. 5a. Comparison by eye shows that the efflux of CF from LA vesicles is much faster than from OA/GMO vesicles, as expected for a more dynamic and unstable type of membrane [16], [17].

Beyond these general qualitative considerations, specific values for the permeability were also calculated. To do this, the previous release data were transformed, following the procedure described in Text S2, and represented in a semi-logarithmic plot (Fig. 5b). Approximately linear trends were obtained for at least the first 30 min of release both for LA vesicles (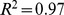) and for OA/GMO vesicles (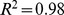), each of the slopes giving the respective rate constant of release 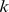, in accordance with Eq. 4 in Text S2 (see Note 6 in Text S1). The corresponding values of 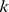 are shown in Table 2 together with other related permeability parameters that can be derived from it. For instance, the permeability coefficients 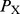 were obtained from the respective values of 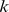, assuming a monodisperse size distribution of unilamellar 400 nm-extruded vesicles (50 nm- in the case of OA/GMO), i.e. 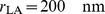; 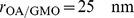 (see Eq. 5 in Text S2). The molecular diffusion coefficients 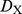 could also be estimated (Eq. 6 in Text S2) taking realistic values for the membrane thickness 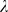 (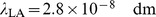; 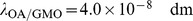) [43]. All this confirms the differences of permeability to CF when LA vesicles and OA/GMO vesicles are compared, the former being manifestly leakier. Notice that even though the permeability was determined from release experiments, similar permeability properties would apply for the entry of CF (see Eq. 1 in Text S2).

**Table 2:** Permeability coefficients calculated from the release of entrapped CF at 45°C.

Composition	pH	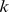 ( 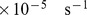 )	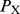 ( 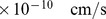 )	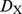 ( 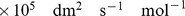 )
LA	7.95	2.60	1.73	2.92
OA/GMO 2∶1	9.10	0.39	0.0328	0.0791

### Permeability Constraints: Time Scale Implications for Compartmentalized Prebiotic Chemistries

Up to this point all processes defined in the protometabolic model have been considered to occur in an arbitrary time scale. This in principle allows for the free assignment of time units, as long as these units are consistent within the different processes taking place in the system. In practice, however, once the identity of the molecules can be established, it will be reasonable to assume certain constraints on the general time scale of events. In fact, efficient catalysts (like protein enzymes) were surely absent in the early stages of the emergence of life, thus inter-conversion rates were also probably much slower in prebiotic conditions; i.e. generally speaking, longer time scales than those characteristic of present biochemistry should be considered. However, even without taking into account how the progressive development of catalysis could accelerate the general pace of protometabolic reactions, permeability values in a compartmentalized system, like ours, will already define important restrictions on the time scale of the encapsulated chemistry. As the maintenance of the system in a functional steady state has been shown to be at risk for relatively low values of the incorporation rate constants (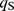, 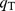 and 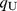), the time scale of the internal protometabolic processes, presumably, cannot be too short (i.e. processes should not occur too quickly). Otherwise, the relatively slow acquisition of food molecules from the environment would not be sufficient to supply the internal demand (see Note 7 in Text S1). For instance, for 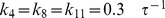 (with a critical value 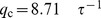), a general time scale of seconds would require values of incorporation rate constants above 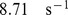, whereas values over 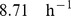 (about 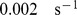) would be sufficient for reactions occurring at a time scale of hours. This observation raises the interesting question of the minimum general time scales that would be compatible with the functioning of this protometabolism in a prebiotic compartment.

Our experimental assays were focused on the permeability to CF, due to its obvious advantages in terms of producing a measurable fluorescence signal. We are aware of the lack of prebiotic relevance for CF and of the fact that vesicle permeability can change quite drastically, depending on the sort of solute entering or exiting, and the actual composition of the membrane [40]. However, CF can be taken as a suitable point of reference for our purposes here: it is a negatively-charged, relatively large molecule, so most nutrients or precursors (at least those that one would expect to be initially necessary for such a primitive metabolic network) should permeate into fatty acid vesicles of a similar composition faster than CF. In other words, in a generalized permeability chart (like the one provided in Fig. 3 of [40]) most molecular species would present higher values than those obtained for CF. Under this assumption and for the sake of simplicity, we will therefore consider that the experimental results with CF can serve as an adequate control (a “less-favorable estimate”) for the passage of prebiotically available organic nutrients through plausible primitive compartment boundaries. But, in any case, the implications, insofar as the non-trivial self-maintenance of the internal reaction network is concerned, will be circumscribed to the particular model–and the particular region of parameter space–explored above.

According to the previous premises, once the diffusion of CF has been experimentally determined, we can substitute the values of 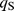, 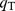 and 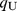 in the protometabolic model by the measured value of 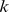 for LA vesicles, first, as the most primitive and precarious case (in other words, 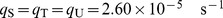). In this way, the restriction on the time scale of the internal protometabolic network can be estimated by comparing 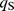, 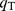 and 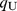 with 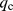. For instance, taking again the condition in which 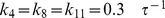, it can be easily shown that neither the time scale of seconds nor the time scale of hours would be long enough to allow the maintenance of the system with relatively high concentrations of intermediates, for 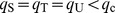 with either unit. Indeed, 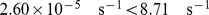, and 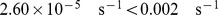. In those circumstances, the general time scale for protometabolic reactions should be longer (i.e. slower processes): it should span at least 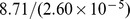 seconds, i.e. 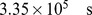. As shown in Table 3, this is first met by a time scale of weeks (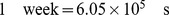). Then, 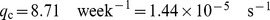 and, as a result, 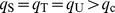. This would imply the restriction that first-order kinetic constants in the model, such as 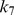, should take at least units of the order of 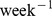 and the second-order kinetic constants, such as 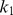, units of 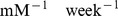.

**Table 3:** Time scales compatible with self-maintenance.

Time scale	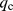 ( 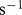 )	Functional in LA vesicles	Functional in OA/GMO vesicles
Second	8.71	−	−
Minute	1.45×10^−1^	−	−
Hour	2.42×10^−3^	−	−
Day	1.01×10^−4^	−	−
Week	1.44×10^−5^	+	−
Month	3.36×10^−6^	+	+

+ Time scales that would allow, in realistic compartmentalized conditions, the maintenance of a functional protometabolism within LA or OA/GMO vesicles, considering the parameter region given by 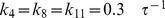 and precursor compounds whose permeability properties would be similar to that of CF.

The analysis to specify or delimit the characteristic reaction time scales of protometabolic processes can be extended to other values of the decay rates 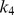, 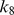 and 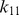. In general terms, we saw that, since the minimum values of trans-membrane diffusion constants required to preserve a functional steady state in the model are located within a narrow range (generally between 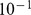 and 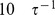), the corresponding critical time scale does not change significantly from one situation to another. In fact, even a hypothetical limit case in which 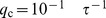 would involve a minimum time scale of days, considering the permeability properties of LA vesicles. The situation becomes more restrictive (in terms of longer time scales–or slower processes–required) if we apply a similar reasoning to the model protometabolism when it is enclosed by OA/GMO vesicles, given the lower permeability of this type of compartment (Table 3). Nevertheless, one should not forget that we are dealing with a “less-favorable estimate” (given by permeability values to CF): many nutrients could cross the membrane more easily than CF and, hence, allow faster processes to occur within the system boundaries.

**Figure 6:**
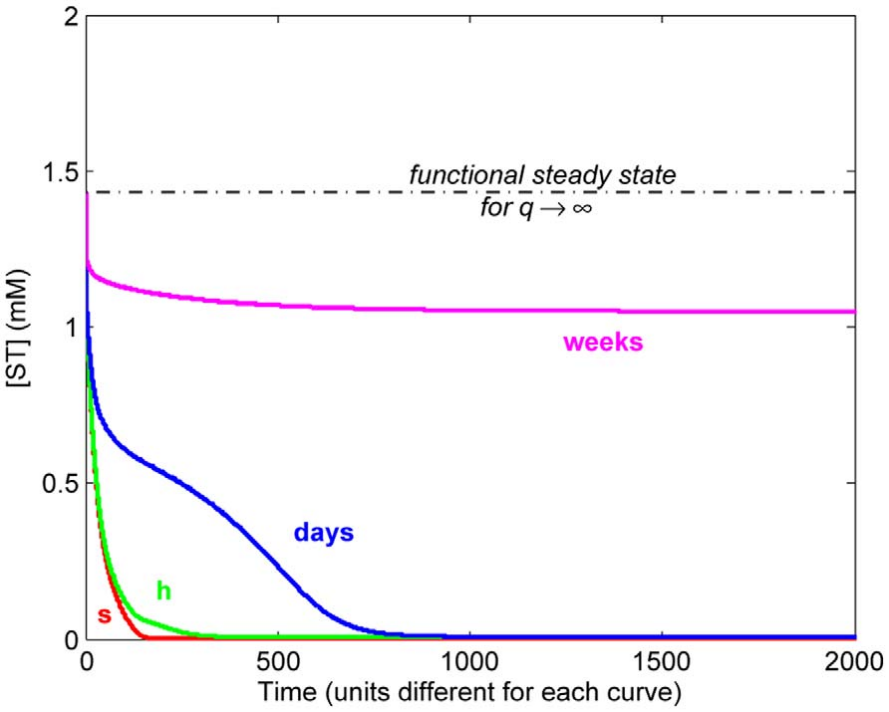
Time behavior of ST concentration. Time evolution of the internal concentration of ST obtained with diverse experiments differing in the time scale of the internal reaction network, i.e. interconversion and degradation rates. In each case, kinetic constants are expressed in units consistent with the respective time scale, e.g. for a time scale of weeks, first-order rate constants have units 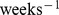 and second-order rate constants have units 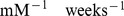. The units in the abscissa axis remain undefined as they are determined by each particular time scale considered. In all cases, 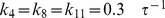 and 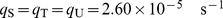.

The actual influence of the time scale on the maintenance of relatively high concentrations of the metabolic intermediates was finally analyzed by numerical integration. Fig. 6 illustrates the time evolution of the concentration of ST for 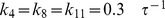 and 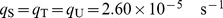 (i.e. assuming that component precursors like CF had to access LA vesicles) for various cases, each with a different time scale for internal protometabolic reactions (including degradation steps). In all of them the initial condition corresponds to those concentrations found at the “functional” (or non-trivial/non-residual) steady state for non-limiting diffusion of precursors (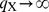). It is shown that, under those conditions, only for a metabolic time scale of weeks does the system end up in a steady state with non-vanishing values of intermediates. These results demonstrate how an adequate relationship between 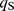, 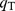, 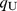 and 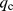 is fundamental to preserve functional concentration levels in the system, confirming that more attention should be given to the chemical constraints involved in compartmentalized prebiotic chemistries, as discussed more extensively below.

## Discussion

Regardless of the specific characteristics of the system explored here (both in terms of the particular type of protometabolic network and membrane/solute permeability duplex), a fairly general conclusion may be drawn from our analysis: it makes little sense to investigate separately the properties of compartments and the chemistries that take place within and around them. From our perspective, it is dangerous to neglect the problem of the encapsulation of complex reaction pathways (regulated through sophisticated catalysts), and to consider their appearance independent from the development of compartments. Indeed, a highly efficient protometabolism (i.e. with enhanced catalytic rates) would most probably perish by self-suffocation, as its need for nutrients and raw materials might not be satisfied by the passive influx of substrates. Conversely, the control of the properties and dynamic plasticity of the boundary of a system becomes really significant when the amphiphilic or lipidic compounds of the membrane are endogenously synthesized (as is the case in biological cells), rather than directly taken from the set of molecules available from the environment. The latter side of the coin becomes more obvious in the context of the transition from *self-assembling* to *self-producing* compartments, analyzed elsewhere [44].

Here we have mainly dealt with the former issue: the viability conditions that compartmentation imposes on reaction networks and, more precisely, with the problem of how membrane permeability to nutrients may limit the characteristic time scale of the protometabolic processes taking place within a prebiotic vesicle. This problem, theoretically posed and discussed in [31], but still lacking a more detailed and specific treatment, has been now addressed with a concrete example. In general terms, we found that even highly permeable compartments, such as LA vesicles, could already entail a remarkable restriction on the time scale of an internally-developing protometabolism, depending on the size and type of compounds that this requires as starting materials. Quite reasonably, these restrictions become more acute as the permeability of the boundary, overall, decreases (e.g. when the membrane is made of more complex or evolved lipid components, like mixtures of OA/GMO). The main corollary of this work, in any case, supports the central idea put forward by Szathmáry [31]: the need to envision a coevolutionary scenario for the development of compartments, hand in hand with the development of catalytic mechanisms. As catalysis becomes increasingly efficient in the system (e.g. through the development of stereospecific molecular recognition mechanisms), compartments should also become increasingly robust and enhance their selective-permeability properties (e.g. through the insertion of specific transport mechanisms that ensure a sufficiently fast entry, across the membrane, of all necessary precursors).

This scenario accords quite well with recent experiments on the relative leakiness of prebiotic, fatty acid compartments [16]–[18], [45], as compared to standard liposomes (biomembrane models) made of more complex amphiphilic molecules, such as phospholipids. Accordingly, the first compartments would be quite permeable to organic compounds of different kinds, imposing relatively “soft” constraints on the possible chemistries within them, but unable to retain within them many of the products of those chemistries (except for the larger compounds). Then, progressively, the lipid bilayer would become less and less permeable, capable of more efficient encapsulation and, in general terms, also more stable. Yet, at the same time as that transition takes place, specific transport mechanism would most probably have to start being developed, among other reasons for the one we have concentrated on in this contribution: the avoidance of a self-suffocating, dead end. In any case, the heterotrophic scenario for the origins of life, favored in principle by Szostak’s group [16], ought to be more carefully assessed, taking into account the specific chemistry of the protometabolic reaction network.

In the present work, the focus has been set on the problem of nutrient accessibility to a compartmentalized protometabolism. We have limited the analysis to a simple, abstract (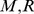)-system [34], considering its interest from a theoretical point of view (catalytic closure ensures the production and maintenance of all catalysts present in the network, a fundamental requirement for metabolism) as well as our previous analysis of this reaction network in open-solution conditions, which confirmed its capacity for robust self-maintenance. Nevertheless, it is necessary to carry out further work on the same general problem using other internal reaction schemes that also support self-production and, whenever possible, take into account the physical-chemical nature of the components involved. In addition, in this first approximation we have disregarded, among other issues, the possible leakage of metabolic intermediates across the membrane, together with the dynamic nature of the compartment itself and the osmotic effects that internally produced species could have, which might turn out to be critical for the global stability of the system. For instance, the rapid accumulation of waste products could lead to an osmotic imbalance, even to a complete disruption of the compartment. We plan to include all these important aspects in future work, since we are aware that they will contribute to gain a better understanding of the early interplay between metabolism and membranes.

## Materials and Methods

### Materials

Lauric acid (LA), oleic acid (OA), bicine, carboxyfluorescein (CF) and Triton X-100 were purchased from Fluka, Switzerland. *rac*-1-Oleoylglycerol (GMO) was obtained from Sigma, USA.

### Preparation of Lipid Vesicles

Vesicle suspensions were prepared by adding the fatty acid, LA, melted in advance, to a preheated 100 mM bicine buffer solution containing 8 mM CF and following the pH vesiculation method [46]: the pH was first increased up to complete transparency of the sample, to maximize the solubility of the oily fatty acid, and then acidified to a pH close to the 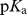 of the fatty acid (see values in Table 2), where vesicular structures become stable. An important point, however, is that samples had to be kept at temperatures over 32°C to avoid precipitation due to the high melting point of LA. A value of about 21 mM for the final total concentration of amphiphile was fixed (well above the critical vesicle concentration or CVC). Mixed systems of OA/GMO were similarly prepared in a 2∶1 molar ratio of OA to GMO (total concentration: 30 mM), but in this case samples could be kept at room temperature.

### Solute Release Measurements

Vesicles were extruded with a small extruder from Avanti Polar Lipids, USA, passing vesicle suspensions (21 times) through polycarbonate filters with either 50 (for the OA/GMO system) or 400 nm (for LA, given its lower stability for smaller sizes) pore diameter. Non-encapsulated CF was removed by size-exclusion chromatography, on an agarose column (Bio-Gel A1.5) previously equilibrated with 100 mM bicine buffer at the corresponding pH. To avoid vesicular disruption during separation, this buffer contained a concentration of amphiphile close to the CVC in the case of LA (for OA/GMO vesicles this was not necessary). 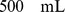 fractions were collected. The fifth fraction was spectrophotometrically and microscopically determined to be the one containing the largest concentration of vesicles. With this fraction, the release of CF was monitored on a spectrofluorometer (Cary Eclipse, Varian) (Ex. 450 nm/Em. 520 nm) at 45°C (above the melting point of LA). A standard curve made from various serial dilution solutions of CF in bicine buffer was used to calculate the release in terms of concentration.

### Computational Analysis and Simulations

A set of ordinary differential equations was defined to describe the system dynamics (see Text S3). Stationary state solutions were obtained by numerical solution of the nonlinear algebraic equations that result from equating the whole set of equations to zero. Solutions found in this way were collected for a wide range of parameter values and eventually presented in the form of bifurcation diagrams.

The stability of each of the stationary states found was checked by means of variational analysis: the Jacobian matrix was evaluated at the particular steady-state values, and the eigenvalues and eigenvectors calculated. Those steady-state solutions having all eigenvalues with negative real parts were identified as asymptotically stable, whereas those with at least one eigenvalue with positive real part correspond to unstable steady states.

Matlab was used for the previously detailed calculations as well as for dynamical simulations, which consisted in experiments of numerical integration of the set of ordinary differential equations and were thus deterministic.

## Supporting Information

Text S1 **Notes from the text.**
(PDF)

Text S2 **Permeability calculation.**
(PDF)

Text S3 **Set of ordinary differential equations of the model protometabolism.**
(PDF)
