# Supplementary material for: Viability Conditions for a Compartmentalized Protometabolic System: A Semi-Empirical Approach
Source: PLoS One. 2012 Jun 27;7(6):e39480. doi: 10.1371/journal.pone.0039480 (PMC3384665; doi:10.1371/journal.pone.0039480)
Supplement: Text S1 — Notes from the text. (PDF) [file pone.0039480.s001.pdf]

Supporting Text S1

Notes from the text

1. The possibility that relatively simple biochemical pathways, such as the synthesis of green fluorescent protein [1], take place within liposomes has been proved experimentally in numerous laboratories, making use of different compounds (not necessarily of prebiotic significance) and diverse techniques (for a review, see [2]). However, to our knowledge, no systematic analysis of the conditions for viability of a compartmentalized protometabolic system has been carried out until now.

2. Mineral surfaces may have played an important role in the prebiotic synthesis of organic compounds that are required for living systems to come about, but the biological way to gain control on diffusion and transport processes—as is obvious from all forms of life known today—was achieved through heterogeneous, compartmentalized domains made of soft, lipid membranes, on which selective permeability mechanisms were implemented.

3. The degradation reactions are assumed to yield non-activated products, which would in turn be eventually released to the environment by passive diffusion through the membrane. However, these species will be disregarded in this work, as they are nonreactive products of irreversible processes, and thus, they do not affect the kinetics of the intermediates considered in the model. Having said that, their transient accumulation in the inner aqueous core could be relevant in more elaborate protocell models that consider features such as osmotic tension and the possibility of osmotic bursting (see Discussion).

4. The model, as analyzed in earlier work [3], was also sensitive to the degradation rate constants, with two main differences. Neglecting the uncatalyzed formation of catalysts meant that there was no stationary state of residual functioning but instead a trivial steady state of null concentrations. Additionally, the region of bistability (i.e. coexistence of trivial and non-null asymptotically stable solutions, separated by an unstable steady state) extended to smaller values of k_4, k_8 and k_{11}. This prevented the recovery or emergence of the system in the absence of catalysts and intermediates, as at least some seeding concentrations were initially required to overcome the separating barrier.

5. The molecular mass of CF is 376 Da, so it is a molecule of intermediate size: small if we compare it with the typical sizes of biologically relevant oligomers, but probably bigger than prebiotic nutrients, or precursors of those oligomers. Another important feature of CF, regarding its permeability through fatty acid vesicles, which carry overall negative charges [4], is that the molecule itself is negatively charged at the pH in which experiments were carried out.

6. The decay curve for LA vesicles is probably higher than first order, so more complex models would be needed to provide a more precise idea of the kinetics of the whole process (see Text S2 for some possible factors altering the dynamics). However, this is not critical in relation to our primary aim here of obtaining an estimate of the initial permeability values.

7. We will assume that all the internal processes keep the relative values of their rate constants according to the parameters given in Table 1 (i.e. ratios are always maintained between them, to ensure similar "rules" and the validity of the bifurcation diagrams), and explore the consequences of varying the time units in which they are expressed. This applies not only to interconversion reactions but also to degradation processes.

References

1. Yu W, Sato K, Wakabayashi M, Nakaishi T, Ko-Mitamura EP, et al. (2001) Synthesis of functional protein in liposome. J Biosci Bioeng 92: 590-593.

2. Stano P, Carrara P, Kuruma Y, de Souza TP, Luisi PL (2011) Compartmentalized reactions as a case of soft-matter biotechnology: synthesis of proteins and nucleic acids inside lipid vesicles. J Mater Chem 21: 18887-18902.

3. Piedrafita G, Montero F, Morán F, Cárdenas ML, Cornish-Bowden A (2010) A simple self-maintaining metabolic system: Robustness, autocatalysis, bistability. PLoS Comput Biol 6.

4. Morigaki K, Walde P (2007) Fatty acid vesicles. Curr Opin Colloid Interface Sci 12: 75-80.
