# Supplementary material for: Viability Conditions for a Compartmentalized Protometabolic System: A Semi-Empirical Approach
Source: PLoS One. 2012 Jun 27;7(6):e39480. doi: 10.1371/journal.pone.0039480 (PMC3384665; doi:10.1371/journal.pone.0039480)
Supplement: Text S2 — Permeability calculation. (PDF) [file pone.0039480.s002.pdf]

# Supporting Text S2

## Permeability calculation

According to the osmotic unbalance, the efflux of solute from the internal aqueous core of the vesicles would be described by the following differential equation:

$$\frac{\mathrm{d}[\mathrm{X}]}{\mathrm{d}t} = -q_{\mathrm{X}}\left([\mathrm{X}] - [\mathrm{X}]_{\mathrm{out}}\right) = \frac{-P_{\mathrm{X}}S}{V}\left([\mathrm{X}] - [\mathrm{X}]_{\mathrm{out}}\right) \tag{1}$$

where [X] and $[\mathrm{X}]_{\mathrm{out}}$ are the internal and external concentrations respectively of solute X; and $q_{\mathrm{X}}$ is the rate constant for the characteristic efflux, or passive diffusion, which can in turn be expressed as $P_{\mathrm{X}}S/V$, where $P_{\mathrm{X}}$ is the permeability coefficient of X across the vesicle membrane, and $S$ and $V$ are the total vesicle membrane surface and total internal aqueous volume, respectively. Integrating the previous expression, the following rule can be derived [1]:

$$[\mathrm{X}]_{\mathrm{out},t} - [\mathrm{X}]_{\mathrm{out},0} = ([\mathrm{X}]_{\mathrm{eq}} - [\mathrm{X}]_{\mathrm{out},0})(1 - e^{-kt}) \tag{2}$$

where $[\mathrm{X}]_{\mathrm{out},t}$ is the external concentration of solute at a time $t$, $[\mathrm{X}]_{\mathrm{out},0}$ the external concentration at the initial time ($t = 0$), and $[\mathrm{X}]_{\mathrm{eq}}$ the equilibrium concentration, which can be experimentally checked after membrane solubilization with a detergent (Triton X-100). The first-order rate constant $k$ has a value approximately equal to the $P_{\mathrm{X}}S/V$ ratio and in practice can be identified with the release rate constant, given its similarity to the parameter $q_{\mathrm{X}}$. This can be obtained easily by calculating the slope from a semi-logarithmic plot of $\ln(([\mathrm{X}]_{\mathrm{eq}} - [\mathrm{X}]_{\mathrm{out},t})/([\mathrm{X}]_{\mathrm{eq}} - [\mathrm{X}]_{\mathrm{out},0}))$ against $t$, as decomposing and rearranging Eq. 2 gives:

$$([\mathrm{X}]_{\mathrm{eq}} - [\mathrm{X}]_{\mathrm{out},0}) - ([\mathrm{X}]_{\mathrm{out},t} - [\mathrm{X}]_{\mathrm{out},0}) = ([\mathrm{X}]_{\mathrm{eq}} - [\mathrm{X}]_{\mathrm{out},0})e^{-kt} \tag{3}$$

which can be linearized by taking logarithms:

$$\ln([\mathrm{X}]_{\mathrm{eq}} - [\mathrm{X}]_{\mathrm{out},t}) = \ln([\mathrm{X}]_{\mathrm{eq}} - [\mathrm{X}]_{\mathrm{out},0}) - kt. \tag{4}$$

The permeability coefficient $P_{\mathrm{X}}$ can be estimated from the slope $k$, assuming a monodisperse size distribution of extruded, spherical vesicles of radius $r$ given the reasonable approximation:

$$P_{\mathrm{X}} \approx \frac{kV}{S} \approx \frac{kr}{3}. \tag{5}$$

Alternatively, we could work with the molecular diffusion coefficient $D_{\mathrm{X}}$, which is related to $P_{\mathrm{X}}$ by the expression:

$$D_{\mathrm{X}} = P_{\mathrm{X}}\lambda N_{\mathrm{A}} \tag{6}$$

where $N_{\mathrm{A}}$ is the Avogadro constant and $\lambda$ the thickness of the membrane.

Note that we are focusing on the *initial* permeability. In other words, we calculate the value of $P_{\mathrm{X}}$ (or $D_{\mathrm{X}}$) from data coming from the first 30 min of the release process. At longer time scales, other phenomena such as aggregation or resizing would take place among these complex structures and could mask the true permeability effect.

# References

1. Chakrabarti AC, Deamer DW (1992) Permeability of lipid bilayers to amino-acids and phosphate. Biochim Biophys Acta 1111: 171-177.
