# Supplementary material for: Viability Conditions for a Compartmentalized Protometabolic System: A Semi-Empirical Approach
Source: PLoS One. 2012 Jun 27;7(6):e39480. doi: 10.1371/journal.pone.0039480 (PMC3384665; doi:10.1371/journal.pone.0039480)
Supplement: Text S3 — Set of ordinary differential equations of the model protometabolism. (PDF) [file pone.0039480.s003.pdf]

Supporting Text S3

Set of ordinary differential equations of the model protometabolism

The time evolution of the concentration of the diverse metabolic intermediates in the model, within the boundaries of the system (see Fig. 1 in the main text) can be described by the following set of ordinary differential equations:

$$\begin{aligned}
\dot{[\mathrm{S}]} &= \bar{v}_{\mathrm{S}} - v_{\mathrm{ST}} - v_{\mathrm{SU}} - v_1 \\
\dot{[\mathrm{T}]} &= \bar{v}_{\mathrm{T}} - v_{\mathrm{ST}} - v_2 \\
\dot{[\mathrm{U}]} &= \bar{v}_{\mathrm{U}} - v_{\mathrm{SU}} - v_{\mathrm{STU}} - v_6 - v_9 \\
\dot{[\mathrm{STU}]} &= v_{\mathrm{STU}} + v_3 + v_7 + v_{10} - v_1 - v_4 \\
\dot{[\mathrm{STUS}]} &= v_1 - v_2 - v_9 \\
\dot{[\mathrm{STUST}]} &= v_2 - v_3 \\
\dot{[\mathrm{ST}]} &= v_{\mathrm{ST}} + v_3 - v_{\mathrm{STU}} - v_5 - v_{11} \\
\dot{[\mathrm{SU}]} &= v_{\mathrm{SU}} + v_7 + v_{10} - v_5 - v_8 \\
\dot{[\mathrm{SUST}]} &= v_5 - v_6 \\
\dot{[\mathrm{SUSTU}]} &= v_6 - v_7 \\
\dot{[\mathrm{STUSU}]} &= v_9 - v_{10}
\end{aligned}$$

in which $\bar{v}_{\mathrm{S}}$, $\bar{v}_{\mathrm{T}}$ and $\bar{v}_{\mathrm{U}}$ are the *net* rates of entry of the three precursors S, T and U from the external reservoir. Each is the product of a generalized flux constant q_{X} and the difference between the external and internal concentration of species X ($[\mathrm{X}_{\mathrm{out}}] - [\mathrm{X}]$) (see Text S2 for further information on permeability features and calculations):

$$\bar{v}_{\mathrm{S}} = q_{\mathrm{S}}([\mathrm{S}_{\mathrm{out}}] - [\mathrm{S}]); \bar{v}_{\mathrm{T}} = q_{\mathrm{T}}([\mathrm{T}_{\mathrm{out}}] - [\mathrm{T}]); \bar{v}_{\mathrm{U}} = q_{\mathrm{U}}([\mathrm{U}_{\mathrm{out}}] - [\mathrm{U}]).$$

The other rates are rates of chemical interconversion, and are assumed to follow mass action kinetics. Thus, according to the terminology in Fig. 1, v_{ST}, v_{SU} and v_{STU} are the rates for the uncatalyzed spontaneous formation of the products ST, SU and STU from their corresponding precursors, and v_i, for $i = 1 \ldots 11$, refer to the rates of the catalytic steps between the different intermediates. For instance, v_1 refers to a reversible transformation process, so it is defined as

$$v_1 = k_1[\mathrm{STU}][\mathrm{U}] - k_{-1}[\mathrm{STUS}].$$

The mass action principle is applied in a similar way to all the other rates. Notice that equations of this kind produce nonlinear terms in the set of ordinary differential equations, and are ultimately responsible for the complex behavior that is described in Results.
